# Editorial: Microbiome: the modulator of human health

**DOI:** 10.3389/fphar.2025.1625412

**Published:** 2025-06-04

**Authors:** Ji Youn Yoo, Moumita Dutta

**Affiliations:** ^1^ College of Nursing, University of Tennessee, Knoxville, TN, United States; ^2^ University of Washington, Seattle, WA, United States

**Keywords:** gut microbiome, bile acid, *Streptococcus*, melatonin, ulcerative colitis, berberine, diabetic cognitive impairment (DCI)

## Introduction

Increasing evidence indicates microbiome plays a crucial role in modulating human health, which influences various pathological as well as physiological processes, including immune and endocrine regulation and neurodevelopment (Jacobson et al., 2020; Zhu et al., 2023; Del Bo et al., 2021). Currently, with the growing emphasis on precision health and multi-omics approaches, the microbiome is increasingly recognized as both a diagnostic marker and a therapeutic target (Yuan et al., 2022; Rosario et al., 2024). This Research Topic brings together cutting-edge studies exploring how the microbiome associates with host health.

## The Microbiome’s power on human disease and therapy: highlights from the Research Topic

One of the most compelling themes emerging from this Research Topic is the dynamic interplay between microbiome and its growing role in both therapeutic interventions and diagnostic applications. The study, Hisamatsu et al. is one of the first to examine the relationship between commonly prescribed medications and alterations in the salivary microbiome among older adults. Notably, statins that are widely used for cardiovascular disease were significantly associated with a reduction in *Streptococcus* abundance. These findings offer a novel perspective on how administered medications may modulate microbial communities beyond the gut, expanding our understanding of host–microbe–medications interactions.

Another study, Zhang et al. introduces a novel diagnostic approach for assessing peritoneal dialysis-related peritonitis (PDRP) risk by utilizing metagenomic next-generation sequencing (mNGS). This advanced technique not only demonstrated higher sensitivity in pathogen detection compared to traditional culture methods but also revealed how variations in dialysis practices influence gut microbial composition. These findings offer valuable insights into the etiological mechanisms of PDRP and highlight the potential of mNGS as a powerful tool for early diagnosis and personalized infection prevention strategies.

An interesting study by Gao et al., depicted the promising therapeutic impact of melatonin on diabetic cognitive impairment (DCI) on murine models. In this study, Gao et al. researchers characterized the promising impact of melatonin in DCI assessing the behavioral parameters, serum levels of inflammatory markers and 16S rRNA sequencing. Wild type (WT) and diabetic (db/db) mice models were used to show the promising therapeutic impact of melatonin treatment in db/db mice. Melatonin treatment ameliorated DCI through the modulation of gut microbiome composition and microbial metabolites. Melatonin treatment enhanced the level of beneficial short chain fatty acid (SCFA) in db/db mice which was considered to reverse the cognitive impairment in the animal group compared to their treatment counterpart.

Yu et al., investigated the ameliorative effects of berberine on ulcerative colitis (UC) models using a multi-omics approach. The authors applied multi omics approach (metagenomics, metabolomics, and transcriptomics) to comprehensively assess how berberine modulates the gut microbiome and bile acids (BA) metabolism to exert therapeutic effects on UC models. A few numbers of beneficial taxa decreased in the gut of UC models and liver produced primary BA enhanced during the experimental period. Berberine not only inhibited the colonization of the pathogenic bacteria but also promoted BA metabolism. The approach opened a new area to explore to understand the therapeutic impact of berberine treatment on UC patients.

## Conclusion and future outlook

The studies featured in this Research Topic highlighted the central role of microbiome in deepening our understanding and improving the treatment of complex diseases. Collectively, these findings strengthen the view that microbial communities are not passive bystanders but active participants in influencing therapeutic outcomes, vulnerability to disease, and the recovery process. Gut Microbiome as well as the microbial metabolites could be an interesting area to modulate host health and host disease development.

As guest editors, we are honored to have curated this diverse and impactful Research Topic. We hope it will encourage further investigation into microbiome-host interactions and will inspire the development of microbiome-informed strategies for personalized health interventions.
